# Enrichment analysis of *Alu* elements with different spatial chromatin proximity in the human genome

**DOI:** 10.1007/s13238-015-0240-7

**Published:** 2016-02-10

**Authors:** Zhuoya Gu, Ke Jin, M. James C. Crabbe, Yang Zhang, Xiaolin Liu, Yanyan Huang, Mengyi Hua, Peng Nan, Zhaolei Zhang, Yang Zhong

**Affiliations:** School of Life Sciences, Fudan University, Shanghai, 200433 China; Banting and Best Department of Medical Research, Donnelly Centre, University of Toronto, Toronto, ON M5S 1A1 Canada; Department of Zoology, University of Oxford, Tinbergen Building, South Parks Road, Oxford, OX1 3PS UK; Institute of Biomedical and Environmental Science & Technology, Department of Life Sciences, University of Bedfordshire, Park Square, Luton, LU1 3JU UK; Department of Bioengineering, University of Illinois at Urbana-Champaign, Champaign, IL 61801 USA; School of Public Health, University of Michigan, Ann Arbor, MI 48109 USA; Department of Molecular Genetics, University of Toronto, Toronto, ON M5S 1A1 Canada; Institute of Biodiversity Science and Institute of High Altitude Medicine, Tibet University, Lhasa, 850012 China

**Keywords:** chromatin interaction, alternative parameter of Hi-C data, open chromatin, methylation potential

## Abstract

Transposable elements (TEs) have no longer been totally considered as “junk DNA” for quite a time since the continual discoveries of their multifunctional roles in eukaryote genomes. As one of the most important and abundant TEs that still active in human genome, *Alu*, a SINE family, has demonstrated its indispensable regulatory functions at sequence level, but its spatial roles are still unclear. Technologies based on 3C (chromosome conformation capture) have revealed the mysterious three-dimensional structure of chromatin, and make it possible to study the distal chromatin interaction in the genome. To find the role TE playing in distal regulation in human genome, we compiled the new released Hi-C data, TE annotation, histone marker annotations, and the genome-wide methylation data to operate correlation analysis, and found that the density of *Alu* elements showed a strong positive correlation with the level of chromatin interactions (hESC: *r* = 0.9, *P* < 2.2 × 10^16^; IMR90 fibroblasts: *r* = 0.94, *P* < 2.2 × 10^16^) and also have a significant positive correlation with some remote functional DNA elements like enhancers and promoters (Enhancer: hESC: *r* = 0.997, *P* = 2.3 × 10^−4^; IMR90: *r* = 0.934, *P* = 2 × 10^−2^; Promoter: hESC: *r* = 0.995, *P* = 3.8 × 10^−4^; IMR90: *r* = 0.996, *P* = 3.2 × 10^−4^). Further investigation involving GC content and methylation status showed the GC content of *Alu* covered sequences shared a similar pattern with that of the overall sequence, suggesting that *Alu* elements also function as the GC nucleotide and CpG site provider. In all, our results suggest that the *Alu* elements may act as an alternative parameter to evaluate the Hi-C data, which is confirmed by the correlation analysis of *Alu* elements and histone markers. Moreover, the GC-rich *Alu* sequence can bring high GC content and methylation flexibility to the regions with more distal chromatin contact, regulating the transcription of tissue-specific genes.

**Electronic supplementary material:**

The online version of this article (doi:10.1007/s13238-015-0240-7) contains supplementary material, which is available to authorized users.

## INTRODUCTION

Transposable elements (TEs) are DNA sequences able to move and replicate within the genome of a single cell and have been found in virtually all eukaryotic genomes so far sequenced (Feschotte 2008; Kidwell and Lisch 2001). Large-scale genome sequencing has revealed that TEs comprise more than 45% of the human genome, and also represent an abundant part of the genomes in fungi and metazoans (3%–20% in fungi and 3%–45% in metazoans) (Wicker et al. 2007; Lander et al. 2001). According to the mechanism of transposition, TEs are usually divided into two classes (Class I or retrotransposons, and Class II or DNA transposons). Retrotransposons can be further grouped into three main orders (long terminal repeats (LTRs), long interspersed nuclear elements (LINE) and short interspersed nuclear elements (SINE)). In humans, *Alu* elements are the most abundant SINEs with over one million inserted copies, resulting from their continuous proliferation activity over the past ~65 million years (Myr) (Cordaux and Batzer 2009), and comprise about 10% of the DNA sequences of the human genome. According to the current model, modern *Alu*s are ~300 bp in length and emerged from a head to tail fusion of two distinct fossil *Alu* monomers (FAMs) that originated from the 7SL RNA gene 55 Myr ago with the expansion of primates (Quentin 1992a, b). During the course of primate evolution, *Alu* elements have spread and formed several distinct subfamilies on the basis of different rates of amplification. The three major subfamilies are: the oldest *Alu*J, intermediately aged *Alu*S, and the youngest *Alu*Y. Previous studies have indicated that *Alu*J and *Alu*S appeared in ancient genome 65–25 Myr, and *Alu*Y 25 Myr (Kapitonov and Jurka 1996). Due to the short evolutionary history of the *Alu*Y class, some *Alu*Y repeats have not went through the purify selection and are also unable to reach the fixation, resulting in the fact that, currently, most of *Alu* elements in the human genome belong to the *Alu*S class.

*Alu* elements, together with other repetitive elements, were originally thought of as genomic parasites and had been long dismissed as selfish or ‘junk’ DNA (Kidwell and Lisch 2001; Brookfield 2005). Accumulating evidence has demonstrated that widespread *Alu* elements are distributed throughout the human genome in a non-random manner (Grover et al. 2003) and play an important role in genome (Kazazian 2004) and gene evolution (Nekrutenko and Li 2001), epigenetic and gene regulation (Feschotte 2008; Lynch et al. 2011; Teng et al. 2011; Polak and Domany 2006). For example, recent studies have indicated that *Alu* elements are predominant in isochores (Hackenberg et al. 2005) and segmental duplications (Jurka et al. 2004). Likewise, the distribution of *Alu* elements was found to have a highly positive correlation with local GC contents and the density of genes or introns (Grover et al. 2004). Moreover Polak et al. demonstrated that the upstream regions of the transcription start site (TSS) are enriched with *Alu* elements that contain many putative binding sites for transcription factors (Polak and Domany 2006). A subsequent study further highlighted that primate-specific *Alu* elements had derived many promoters and were thus considered to contribute a lot to lineage-specific patterns of gene expression in humans (Huda et al. 2011). In addition, except for the involvement of transcriptional regulation, several lines of evidence have shown that *Alu* elements also play a role in the post-transcriptional regulation by deriving target sites for miRNAs in the 3′ untranslated region (UTR). For example, Jordan and colleagues illustrated that nearly 20% of the human genes contain TEs in the 3′ UTR (Jordan et al. 2003), and it was also observed that some *Alu* elements within the 3′ UTR of human mRNAs are highly conserved and provide perfect complementary target sites for miRNAs (Smalheiser and Torvik 2006).

Transcription of eukaryotic genes is a highly complicated process requiring the ultra-precise cooperation of a batch of interactions among functionally diversified proteins and DNA sequences (Maston et al. 2006). Regulation of transcription is performed by a myriad of *cis*-regulatory elements, including enhancers, promoters, silencers and insulators, and fulfilled mainly by enhancers, which are portions of DNA that can activate transcription regardless of their location, distance or orientation relative to the promoters of genes by binding a variety of transcription factors (Ong and Corces 2011). This functional flexibility has indeed diversified the transcriptional regulation in different tissues or cell types. For example, a recent study examined the genome-wide histone H3 lysine 4 (H3K4) methylation patterns in two cancer cell lines—K562, a human erythroleukemia, and HeLa, a human cervical carcinoma, and predicted about 24,000 to 36,000 enhancers in each cell line, and found that only 5000 (14%–21%) are present in both, indicating the high cell-type-specificity of enhancer activity (Heintzman et al. 2009). Nevertheless, this flexibility impedes efforts to comprehensively investigate and record the full list of enhancers and promoters they regulated within the genome.

The systematic identification of a high-resolution *cis*-regulatory map between enhancers and their target promoters in mammalian systems has so far been limited. However, recently, 3C (chromosome conformation capture) based technologies have enabled large-scale spatiotemporal interactions, between distal sequence elements (mainly enhancers and promoters) within genomic loci located throughout the mammalian genome, to be routinely investigated by high-throughput microarray or deep sequencing approaches (Lieberman-Aiden et al. 2009; Wang et al. 2011; Eskeland et al. 2010; Kagey et al. 2010). 3C-based protocols were originally used to evaluate long-range chromatin interactions (CIs) between a pair of pre-specified genomic sites (Dekker et al. 2002). The extensions of the 3C approach include 4C (chromosome conformation capture-on-chip) (Simonis et al. 2006), 5C (chromosome conformation capture carbon copy) (Dostie et al. 2006), ChIA-PET (Chromatin Interaction Analysis with Paired-End Tag) (Fullwood et al. 2009) and Hi-C (Lieberman-Aiden et al. 2009) (reviewed in de Wit and de Laat 2012). Among these methods, Hi-C allows the detection of chromatin interactions between any pair of loci across an entire genome at a higher resolution (Lieberman-Aiden et al. 2009). In Hi-C experiments, CIs can be determined by the number of Hi-C reads and the read count is negatively correlated with the three-dimensional (3D) distance between two loci, suggesting that Hi-C read count can serve as a powerful proxy to measure the CI level (Lieberman-Aiden et al. 2009). Recently, Dixon et al. reported an investigation of the 3D organization of the human and mouse genome in four different cell types at unprecedented resolution using Hi-C method (Dixon et al. 2012) and identified large, mega-base-sized local chromatin interaction domains, termed “topological domains”, as a pervasive structural feature of the genome organization in human and mouse. Such a pervasive structural characteristic was subsequently confirmed by an independent study (Nora et al. 2012). Further investigation reveals that the overall domain structure is generally conserved during mammalian evolution, and largely stable between different cell types, whereas intra-domain contributes most (>96%) of dynamic interacting regions, and thus potentially participate in cell-type-specific regulatory events (Dixon et al. 2012). Furthermore, they also observed that *Alu*/B1 and B2 elements in mice and *Alu* elements in humans are enriched at boundary regions of domains, indicating a role for TEs in the spatial organization of the genome. In contrast to the intense research on the spatial characteristics of TEs at boundary regions, TEs themselves, as the most abundant part in mammalian genomes, many unanswered questions associated with their function and involvement in intra-domains have largely remained uncharacterized. For example, whether TEs actively take part in the cell-type-specific regulation in intra-domains, and if so, how, is still mysterious.

To address these issues, in this study, we used newly released, genome-wide chromatin interaction data provided by Dixon et al. (Dixon et al. 2012) to investigate the possible roles for TEs, especially *Alu* elements, in intra-domain regions, and further link the TEs with EP interactions in human. First, we explored the association between TEs and CIs. Our results showed that SINE coverage is positively correlated with CI frequency in both pluripotent and differentiated cell lines, while other TE families are not. Further analyses indicated that such positive correlation is solely contributed by *Alu* elements. Second, we investigated whether the positive correlation between *Alu* coverage and CI frequency is due to the involvement of *Alu* elements in enhancer-promoter interactions. We found that enhancers or promoters are increased during the growing of CI frequency, resulting in the increasing of their possible interaction pairs. We then studied the relationship between *Alu* coverage and enhancer-promoter interactions, and found they indeed had a significantly positive correlation in different cell types, which indicated that the amount of *Alu* elements may be an alternative parameter to evaluate the distal interaction between enhancers and promoters. To test whether this pattern holds true in the human genome, we validated these results using a data set coming from an independent study. The examination confirmed our result to be a general feature in the human genome independent from tissue type.

Besides deriving enhancers and being involved in enhancer-promoter interaction, the *Alu* elements also play an important role in providing potential methylation sites. Previous studies showed that G or C nucleotides will accumulate in regions with more chromatin interactions (Dostie et al. 2006). And the *Alu* elements, especially the old *Alu*J and *Alu*S, have been known to be enriched in GC-rich regions ever since the sequencing of the human genome (Lander et al. 2001). Our results not only prove the phenomenon found in old studies again, but also for the first time showed that the *Alu* elements may be derived from increasing of GC content in high-CI regions, and CpG sites also, which may raise the methylation potentiality as a result. The high-CI regions are reported to be associated with open chromatin (Smit 1999) and are highly related to tissue-specific transcription by the regulation of distal DNA elements, nucleosome modification, and so on. The *Alu* elements can be therefore involved in tissue-specific regulation by raising methylation flexibility and deriving binding sites for transcriptional factors.

Taken together, our results show for the first time that *Alu* elements play a role in chromatin interactions, and its coverage can be served as a surrogate of CI frequency and be used to detect potential enhancer-promoter interaction. Additionally, the more *Alu* elements in regions with more chromatin contacts will endow the regions with methylation capacities and flexibilities, and may be important for tissue-specific transcription. This means that the spatial characteristics of TEs may play a more important role in conformation of chromatin structure and gene activity.

## RESULTS

### SINE elements are over-presented in the genomic loci of intra-domains with high frequencies of chromatin interactions

The recently released whole-genome CI profiling data in mammals include human embryonic stem cells (hESCs), human IMR90 fibroblasts, mouse embryonic stem cells and mouse cortex (Dixon et al. 2012), among which here we focused our analysis on two human cell lines. Several lines of evidence have previously demonstrated that the majority of functional *cis*-regulatory interactions occur within the same topological domain (Dixon et al. 2012; Nora et al. 2012). For example, although the distance between the Shh enhancer and the target shh gene exceeds nearly 1 Mb, they still locate within the same topological domain (Smallwood and Ren 2013). The frequency of intra-domain CIs is higher than inter-domain CIs and a higher frequency means a higher probability to be a real interaction (Li et al. 2010), suggesting that CIs between two genomic loci, located at different domains, may be largely non-informative or background noise. In other words, frequency of inter-domain CIs could serve as an effective threshold to remove false-positive CIs. Based on this assumption, we filtered the frequency of intra-domain CIs using mean frequency of inter-domain ones as a threshold, and removed the bin-pairs whose frequencies are lower than the threshold in each of the two human cell lines (see MATERIALS AND METHODS). Furthermore, it was also observed that bin-pairs with extremely high frequencies are scattered, indicating them as possible outliers. We subsequently discarded the bin-pairs whose frequencies are larger than the thresholds of 153 and 133 in hESCs and IMR90 fibroblasts, respectively. The upper thresholds were chosen according to the following reasons: (1) they are the first window without interacting bin-pairs in each cell lines, when we binned the frequency of intra-domain CIs by the step of 1; (2) after applying the upper threshold in each cell line, the removed bin-pairs only occupied 0.0085% and 0.0076% of valid ones in human ESCs and IMR90 fibroblasts, respectively (Fig. S1). Finally, we ambiguously retained 897,867 and 850,305 intra-domain CI bin-pairs (hereafter referred to as CI-bin-pairs, unless otherwise mentioned) in hESCs and IMR90 fibroblasts, respectively.

**Figure 1:**
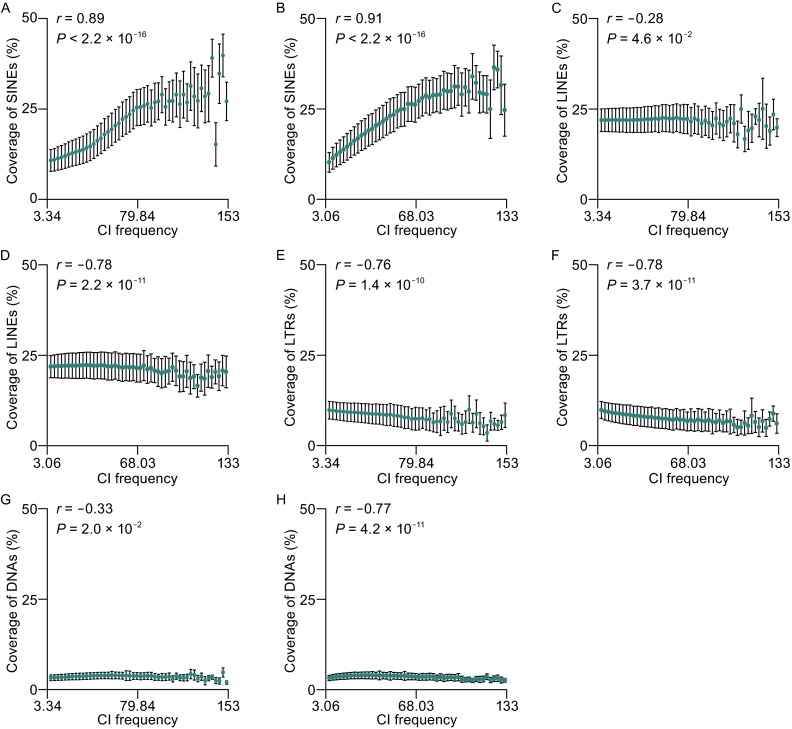
**Mean coverage (MC) of four TE types in each 50 frequency bin**. (A) The MC of SINE in 50 frequency bins in hESC. (B) The MC of SINE in 50 frequency bins in IMR90. (C) The MC of LINE in 50 frequency bins in hESC. (D) The MC of SINE in 50 frequency bins in IMR90. (E) The MC of LTR in 50 frequency bins in hESC. (F) The MC of SINE in 50 frequency bins in IMR90. (G) The MC of DNA in 50 frequency bins in hESC. (H) The MC of SINE in 50 frequency bins in IMR90

We first sought to examine whether a similar distribution pattern of intra-domain CI frequency (hereafter referred to as CI frequency, unless otherwise mentioned) exists in four different TE families. To do this, we divided CI frequencies into 50 bins (referred to as frequency bin), and computed density for each of the four TE families in each frequency bin. The density is the fraction of TEs belonging to the same family in one CI-bin-pair, divided by the maximum possible same-family TEs, which is the ratio of the length of each CI-bin-pair (80 kilobases) to the mean length of that TE family (Fig. 1). The density was calculated for each of the four TE families and plotted across different bins in hESCs and IMR90 fibroblasts, respectively (see Table S1 for TE families with calculated density in each cell line). Higher density implies that more same-family TEs are in a frequency bin, whereas a lower one means less same-family TEs. We noticed that two TE families, LTR and DNA transposons, have comparable low densities, yet SINE and LINE have higher densities across different bins in both cell lines (Fig. 1). This phenomenon may be due to the reason that DNA transposons and LTRs either are currently not mobile or have a very limited activity in the human genome compared to SINEs and LINEs, where their subfamilies, such as *Alu*s and L1s, are still active in humans and have proliferated during primate evolution (Cordaux and Batzer 2009). Then, the Pearson correlation coefficient (PCC) score was computed for each of four different TE families in each cell type, and we found that SINE density is positively correlated with CI frequency in hESC (*r* = 0.89, *P* < 2.2 × 10^−16^, Fig. 1A), and this trend for SINEs is also observed in IMR90 fibroblasts (*r* = 0.91, *P* < 2.2 × 10^−16^, Fig. 1B). But the densities of other three TE families (LINE, LTR, and DNA) are negatively correlated in both cell lines (Fig. 1C–H). To evaluate whether the above results will be affected by bin lengths, we further separated CI frequency into 20 and 100 bins, respectively. The results illustrated that the trends of different TE families, previously observed in the bin size of 50, were still hold true for the bin sizes of 20 and 100 (Figs. S2 and S3).

Since the above results are not affected by the different bin sizes, we then tested whether these results are significantly different from those of non-intra-domains. The correlation between density and CI frequency in non-intra-domains were subsequently calculated for each of the four TE families, respectively. We found that no TE families are correlated with interaction frequency in non-intra-domains, except for DNA transposons in IMR90 fibroblasts with a weakly negative correlation (*r* = −0.377, *P* = 0.013, Table S1). We further compared the correlations of DNA transposon between intra- and non-intra-domains using a z-test, and found that they are significantly different (*P* = 0.0016). Thus, these results clearly demonstrate that the density of four TE families in intra-domains are significantly different from those in non-intra-domains, and SINE is the only TE family whose distribution shows strong correlations with the frequency of intra-domain CIs in both investigated cell lines. Such correlation is also supported by a recent study, in which they indicated that multigene chromatin interacted regions are enriched with SINE density.

### *Alu* is the major player of TEs in intra-domain chromatin interactions

**Figure 2:**
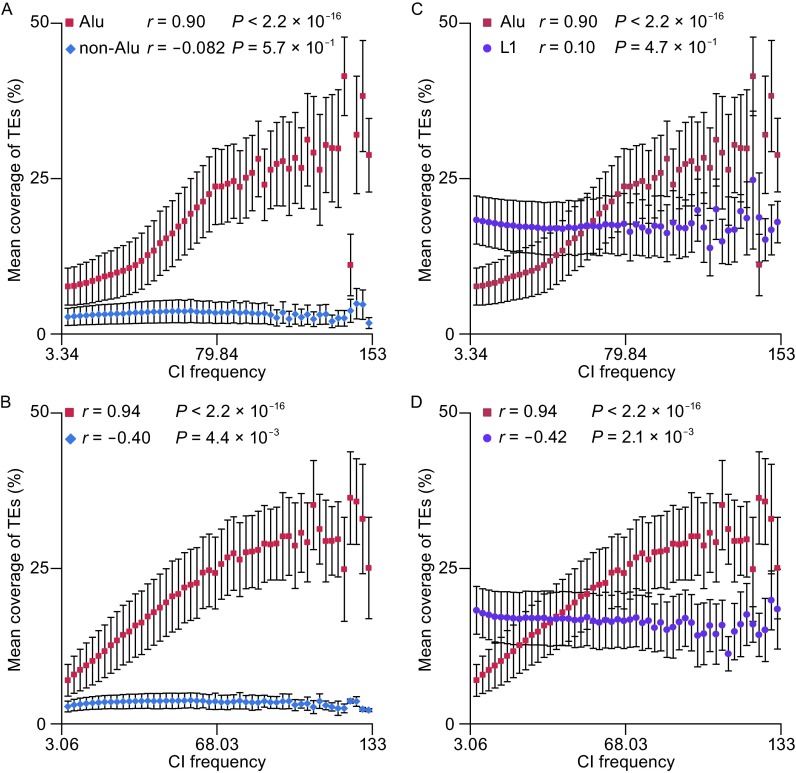
**MC of SINE/** ***Alu*** **, SINE/non-** ***Alu*** **, and LINE/L1 in each frequency bin**. (A) In hESC, the MCs of *Alu* were positively correlated with CI frequencies (*r* = 0.90, *P* < 2.2 × 10^16^), but the MCs of SINE/non-*Alu* were not correlated with CI frequencies (*r* = −0.082, *P* = 0.57). (B) Although the MCs of *Alu* were also positively correlated with CI frequencies in IMR90 fibroblasts (*r* = 0.94, *P* < 2.2 × 10^16^), the MCs of SINE/non-*Alu* in IMR90 fibroblasts were negatively correlated with CI frequencies (*r* = −0.40, *P* = 0.0044). (C) Different patterns of the correlation of the MCs of *Alu* and L1 with CI frequencies were observed in hESC, with a positive correlation of *Alu* and no correlation of L1 (*r* = 0.11, *P* = 0.47). (D) In IMR90 fibroblasts, on the contrary of the positive correlation of the MCs of *Alu*, the MCs of L1 were negatively correlated with CI frequencies (*r* = −0.42, *P* = 0.002)

As *Alu*s are the most successful active TEs in the human genome in terms of copy number, and occupy two-thirds of the SINE elements, this prompted us to contemplate whether *Alu*s contributed the most to the correlation between SINE density and CI frequency in intra-domains. To prove this hypothesis, we divided SINE elements into two subgroups, *Alu*s and SINE/non-*Alu*s, and investigated them separately. Compared with SINE/non-*Alu*s, we found that *Alu* density showed very strong correlations with CI frequencies in both hESC (*r* = 0.90, *P* < 2.2 × 10^−16^) and IMR90 fibroblasts (*r* = 0.94, *P* < 2.2 × 10^−16^), in sharp contrast with PCC scores of −0.082 (*P* = 0.57) and −0.4 (*P* = 0.0044) for SINE/non-*Alu*s in hESC and IMR90 fibroblasts, respectively (Fig. 2A and 2B). Likewise, we noticed that SINE/non-*Alu*s are even not associated with CI frequencies due to the insignificant *P*-value (*P* > 0.05). The difference between *Alu*s and SINE/non-*Alu*s may be due to the reason that, except for *Alu*s, most of SINEs have lost their mobilization activity in mammals, and such a strong correlation between *Alu* density and CI frequency in both investigated cell lines is consistent with our assumption that *Alu*s are the major contributors of SINE elements in intra-domain interactions.

As mentioned previously, *Alu*s can be grouped into three major groups (old *Alu*J, middle *Alu*S, and the young *Alu*Y) that emerged at different time points during the course of primate evolution. We further ask whether a similar tendency can be observed in different *Alu* subfamilies. To do this, we investigated the characterizations of each *Alu* subfamilies by calculating their density across different frequency bins. The distribution of density of three subfamilies in two cell types was plotted in Fig. S4. The densities of three major subfamilies are all positively correlated with CI frequencies in both hESC and IMR90 fibroblasts, suggesting that they have similar distribution tendency in intra-domain interactions.

Considering that *Alu*s are active TEs in the human genome, we doubted whether other active TE families in the human genome shared a similar pattern as *Alu*s. Besides *Alu*s, there are two other TE subfamilies, L1 and SVA, which can actively transpose in humans (Chen et al. 2005). Since SVA is very sparse in the human genome with only 1750–3500 copies, we only focused on the L1 family by exploring the relationship between L1 density and CI frequency. Fig. 2C and 2D clearly demonstrated that L1 densities are not correlated with CI frequencies in hESC (*r* = 0.11, *P* = 0.47), and negatively correlated in IMR90 fibroblasts (*r* = −0.42, *P* = 0.0021). Although L1s and *Alu*s are both active in the human genome, they show no similar distribution patterns in each of the two cell lines. Such a huge difference between them may be due to the reason that *Alu*s have a greater tendency to be retained in the genomic loci with higher frequency of intra-domain interactions, whereas L1 elements do not or even tend to deplete in the higher frequency loci, although both of them maybe integrate into genome randomly. Our finding strongly indicated that more than 81% (*r*^2^) of the variation in *Alu* density could be explained by CI frequency, suggesting that *Alu*s may play an important functional role in intra-domain interactions. Taken together, our results strongly emphasized that *Alu*s are over-presented in genomic loci of intra-domains with high CI frequencies.

### Abundant *Alu*s in highly interacted CI regions contribute more GC content and CpG sites, and probably facilitate to maintain an open chromatin status

The SINE elements are long known to be appeared in GC-rich regions that are associated to open chromatin (Smit 1999). Previous studies have indicated that chromatin interactions are enriched in higher GC-rich regions (Li et al. 2012), so as the SINE elements (Korenberg and Rykowski 1988). However, it is still a mystery what relationships there are among CI frequency, GC content and SINE density.

**Figure 3:**
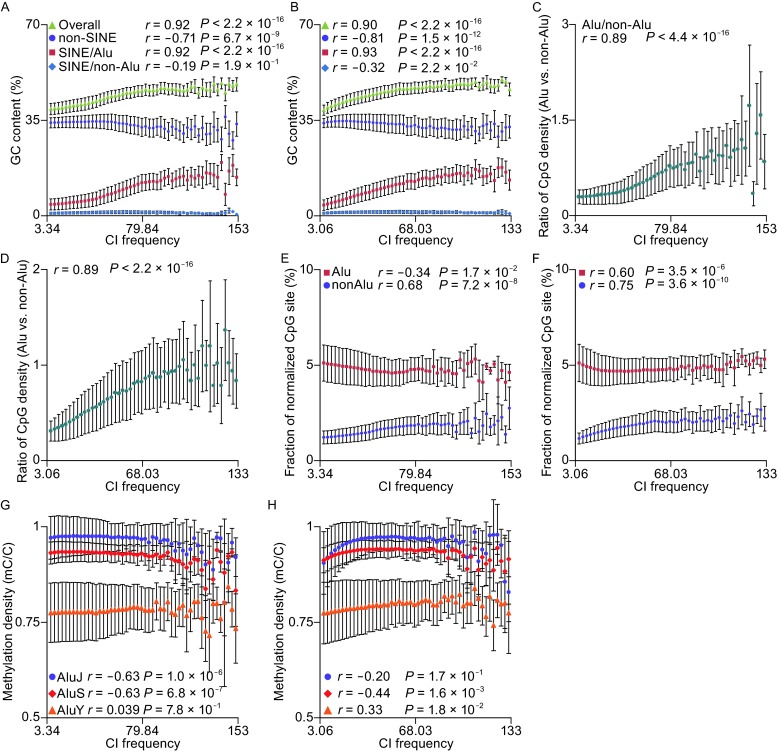
**The GC content, CpG content, and methylation rate of bin-pairs and SINEs**. (A and B) GC content of genome background, *Alu* elements regions, SINE/non-*Alu* regions (other transposable elements in SINE type) and non-SINE regions (regions that aren’t covered by SINEs) in each frequency bin (A: hESC; B: IMR90 fibroblasts). (C and D) The ratio of CpG density of *Alu* regions and genome non-*Alu* regions (*Alu* regions + SINE/non-*Alu* regions + non-SINE regions) increased as the increasing of CI frequencies. (C: hESC; D: IMR90 fibroblasts). (E and F) The fraction of normalized CpG sites in *Alu* regions and non-*Alu* regions (*Alu* regions + SINE/non-*Alu* regions + non-SINE regions) (E: hESC; F: IMR90 fibroblasts). (G and H) Methylation density (the ratio of the methylated cytosine in CpG site) of three *Alu* subfamilies, *Alu*J, *Alu*S, and *Alu*Y (G: hESC; H: IMR90 fibroblasts)

To answer this question, we first divided total GC content (background) in each CI-pair into three major groups (*Alu*, SINE/non-*Alu* and non-SINE) according to their origins, and then calculated the arithmetic mean of GC content values for each CI frequency bin. We found that high CI frequencies are significantly enriched in GC-rich regions in both hESC (*r* = 0.92, *P* < 2.2 × 10^−16^, green triangle) and IMR90 (*r* = 0.9, *P* < 2.2 × 10^−16^, green triangle), which is consistent with the previous study (Li et al. 2012) (Fig. 3A and 3B). Interestingly, further investigation indicates that the GC content contributed by *Alu*s are significantly correlated with CI frequencies in hESC (*r* = 0.92, *P* < 2.2 × 10^−16^, red square) and IMR90 (*r* = 0.93, *P* < 2.2 × 10^−16^, red square), whereas those contributed by SINE/non-*Alu*s are not or negatively correlated with CI frequencies in hESC (*r* = −0.19, *P* = 0.19, blue diamond) and IMR90 (*r* = −0.32, *P* = 0.022, blue diamond), respectively (Fig. 3A and 3B). On the other hand, GC contents derived from *Alu*s, on average, are generally higher than those from SINE/non-*Alu*s, suggesting that large proportion of GC contents in SINE elements are contributed by *Alu*s. We also observed that there are strong negative correlations between GC contents contributed by non-SINEs and CI frequencies in both cell types (*r* = −0.71, *P* = 6.7 × 10^−9^ for hESC; *r* = −0.81, *P* = 1.5 × 10^−12^ for IMR90, Fig. 3A and 3B). These results demonstrated that the enrichment of CI frequencies in GC-rich regions is solely due to the increased copies of *Alu* elements, suggesting that enrichment of *Alu* elements in high CI frequency loci may be not just a result of higher GC contents but a cause of it. We concluded that the tendency toward overrepresentation of *Alu*s in high CI frequency regions maybe due to important functional roles in the formation of chromatin structure and transcriptional regulation.

Higher GC content can not only stable the DNA structure, but also provide more CpG sites for methylation. In mammals, cytosine methylation is, currently, the only covalent DNA modification and restricted to CpG sites. CpG islands are genomic regions that contain a high frequency of CpG dinucleotides, and commonly represent promoters, which are usually located in GC dense regions. Recent research has demonstrated that strong CpG island promoters are mostly unmethylated, even when inactive, whereas CpG poor or weak CpG island promoters are largely hypermethylated, or are preferential targets for *de novo* methylation in human, respectively (Weber et al. 2007). This result indicates that CpG islands with CpG sites tend to be hypomethylated to allow an open chromatin organization and facilitate neighboring gene expression. As *Alu*s encompass ~25% of all CpG sites in the human genome, Xie et al. investigated the epigenetic status of CpG sites of *Alu* elements in human ependymomas and found that methylation status of the majority of CpG sites within or adjacent to *Alu* elements remained unchanged, and differentially methylated CpG sites, in normal control and ependymomas, are enriched in the loci with low CpG density in the flanking regions of *Alu* elements, rather than within the *Alu* sequences themselves (Xie et al. 2010), suggesting CpG sites within *Alu* elements may be more resistant to alteration in methylation than those in the flanking regions. Based on this evidence, we then asked whether the increased GC contents contributed by *Alu*s in high CI frequency loci result in elevated CpG density and further function in maintaining an open chromatin structure for highly active CIs. To tackle this question, we analyzed CpG density contributed by three above-mentioned categories (*Alu*, SINE/non-*Alu* and non-SINE) and compare them with background (see MATERIALS AND METHODS). We found that background CpG density is correlated positively with CI frequency (*r* = 0.90, *P* < 2.2 × 10^−16^ for hESC, Fig. S5A; *r* = 0.95, *P* < 2.2 × 10^−16^ for IMR90, Fig. S5B). Similar to GC content, strong correlations between CpG density contributed by *Alu*s and CI frequency were observed (*r* = 0.88, *P* < 2.2 × 10^−16^ for hESC, Fig. S5A; *r* = 0.95, *P* < 2.2 × 10^−16^ for IMR90, Fig. S5B). In contrast, CpG density contributed by SINE/non-*Alus* is not correlated with CI frequency and, on average, is much lower than that from *Alus*, suggesting that the majority of CpG sites contributed by SINEs come from *Alu* elements. We then performed the correlation analysis comparing the CpG sites provided by *Alu* sequence and the rest sequence termed “non-*Alu* sequences”. The CpG sites provided by *Alu* sequences increase significantly faster than the non-*Alu* sequences as the increase of CI frequencies (Fig. 3C and 3D). We then looked into the proportion of how much the GC content becoming CpG sites; the results showed that the proportion of CpG sites in the GC nucleotide in *Alu* elements is stable as the increase of CI scores, but is increased in the region not covering by *Alu* elements (Fig. 3E and 3F). This suggests to the increase of GC content and CpG sites may be connected with the growth in *Alu* number, but not the changing of the *Alu* sequences. The following analysis of the methylation level of the CpG sites in *Alu* elements showed that older *Alu* elements have higher methylation level than younger ones (Fig. 3G and 3H).

Our result demonstrated that the increased parts of CpG sites in higher CI frequency loci are mainly contributed by *Alu*s, which is consistent with our assumption. In summary, our analysis strongly suggested that, in human, GC content that is enriched in higher CI frequency loci are mainly due to the tendency towards overrepresentation of *Alu* elements and further significantly increased CpG density in these regions. Such elevated CpG density may protect the regions from methylation and allows an open chromatin structure to increase binding probability of ubiquitous transcription factors (Deaton and Bird 2011).

### *Alu*-derived Enhancers and Promoters are significantly enriched in higher CI frequency regions

According to our results, *Alu* coverage showed strong positive correlation with CI frequency (Fig. 2), highlighting that the enrichment of *Alu*s in high CI frequency regions will leads to elevated GC contents and CpG sites that might function in maintaining an open chromatin structure to increase the binding probability of transcriptional factors (Fig. 3A–D). However, such enrichment will also evolve in distal DNA elements regulation directly, like deriving enhancers and promoters that forming distal interactions. Previous studies indeed supported this hypothesis that enhancers are enriched in TE-derived genomic sequences. For example, an *Alu*-containing enhancer regulates the *CD8a* gene in human T cells (Hambor et al. 1993). Likewise, promoter regions are also hotspots of TEs that provided alternative promoters or binding sites for transcription factor (TF). For instance, the LTR-derived promoter significantly increases the expression of human EBR gene in placenta (Medstrand et al. 2001), and *Alu* elements provide binding motifs for three zinc-finger TFs (Sp1, estrogen receptor alpha, and YY1) (Oei et al. 2004). Because chromatin interaction is usually used to exam enhancer-promoter interactions, we determined to investigate the relationship between enhancer-promoter interactions and CI frequency using histone modification marks. Since active enhancers are marked by mono-methylation of H3 lysine 4 (H3K4me1) together with acetylation of H3 lysine 27 (H3K27ac), and active promoters are associated with tri-methylation of H3 lysine 4 (H3K4me3), we analyzed the data downloaded from the UCSD Human Reference Epigenome Mapping Project (see MATERIALS AND METHODS). We first computed the expression levels of H3K4me3-marked genes, and found that these genes indeed expressed higher than non-active ones (Fig. S6), which is consistent with the previous evidence (Santos-Rosa et al. 2002; Santos-Rosa et al. 2003; Schneider et al. 2004).

**Figure 4:**
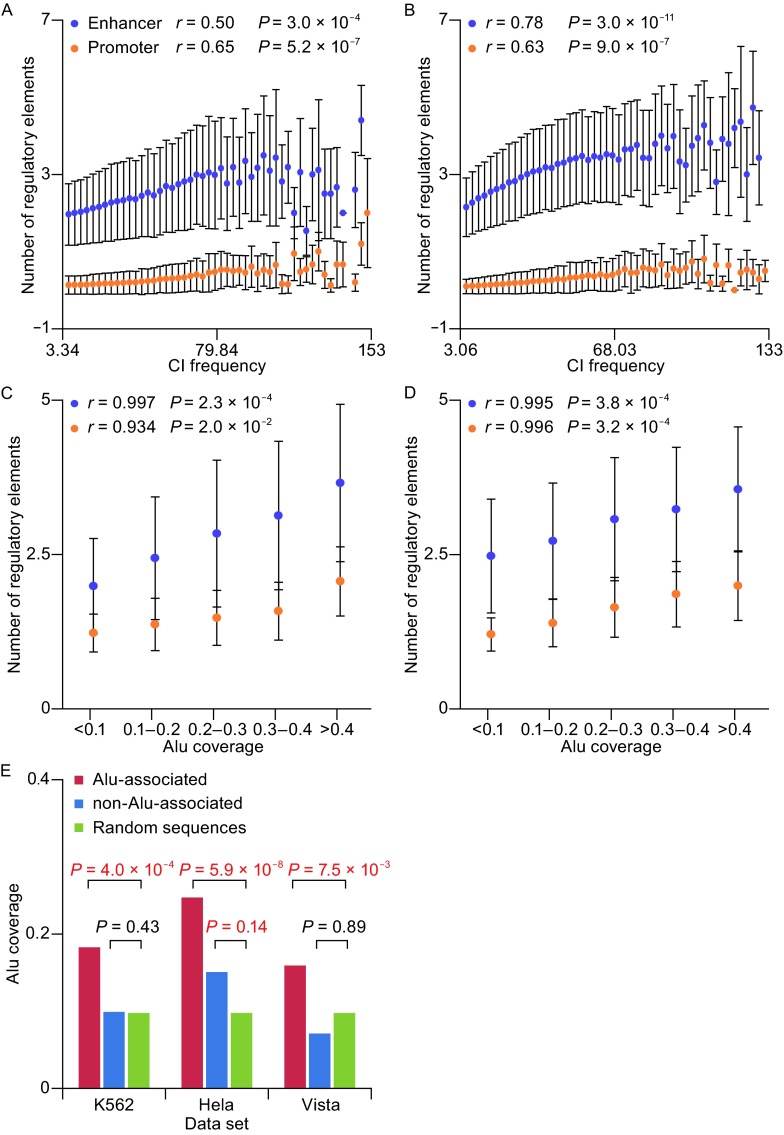
**Mean numbers of enhancers and promoters demonstrated correlation with CI frequencies**. (A and B) The mean numbers of active enhancer regions and active TSS regions were both positively correlated with CI frequencies in two cell lines (A: hESC; B: IMR90 fibroblasts). (C and D) The mean numbers of active enhancer regions and active TSS regions were both positively correlated with *Alu* coverage of the bin-pairs they belonged to in two cell lines (C: hESC; D: IMR90 fibroblasts). (E) The *Alu* coverage in the 40 kb regions centered on the predicted enhancer peaks of K562 cell line, HeLa cell line, and in VISTA enhancer browser. The *Alu*-overlapped enhancers in the two cell lines and VISTA enhancer browser were all significantly different from the background, which is the mean *Alu* coverage of a random 40 kb region in human genome (Binomial test, *P* < 0.01), and the non-*Alu*-overlapped enhancers in HeLa cell line were also significantly different from the random distribution (Binomial test, *P* < 0.05)

After mapping active enhancers and TSSs to the chromatin interaction bin-pairs, we found that the number of enhancers and TSSs are positively correlated with CI frequencies in both hESC and IMR90 cell lines, indicating that the enhancers and promoters are enriched in the interacted genomic loci with high CI frequencies (Fig. 4A and 4B). Since *Alu* coverage increased with growing CI frequency, we asked whether *Alu* elements contribute a lot to the increasing trends of enhancers and promoters during the increase of CI frequencies. To answer this question, we divided the genome into 40 kb bins and assigned each bin to 5 groups based on the Alu coverage of the sequence. We then compared the distribution of regulatory elements in the bins of the 5 groups. By correlation analysis of the coverage of *Alu* elements and the number of enhancers and TSSs, we found that both the enhancers and TSSs showed striking positive correlation with *Alu* coverage (Enhancer: hESC: *r* = 0.997, *P* = 2.3 × 10^−4^; IMR90: *r* = 0.934, *P* = 2 × 10^−2^; Promoter: hESC: *r* = 0.995, *P* = 3.8 × 10^−4^; IMR90: *r* = 0.996, *P* = 3.2 × 10^−4^, Fig. 4C and 4D), suggesting the *Alu* coverage can partially indicate the enrichment of active enhancers and TSSs. This result was confirmed by further examination by using two other independent data sources, the experimental reviewed enhancers in VISTA enhancer browser, and predicted enhancers of K562 and HeLa cell lines predicted by Heintzman ND et al. (Heintzman et al. 2009). We grouped the enhancers based on whether they are covered by *Alu* elements, and analyzed the 40 kb sequence centered on the enhancers. The results demonstrated that the regions around *Alu*-derived enhancers have significantly high *Alu* coverage compared with the randomly selected regions in both two cell lines and VISTA dataset (*P* < 0.05). The result was verified in flanking regions of the enhancers of different length (Fig. S8). The enrichment of active enhancers and TSSs in regions with more distal chromatin interaction and higher *Alu* coverage showed that *Alu* elements may be able to predicted distal enhancer-promoter interaction as a complement of Hi-C data, and may have some biological function in distal regulation of gene transcription.

## DISCUSSION

### *Alu* elements are highly evolved in genome-wide chromatin interaction

*Alu* elements, the active SINE in human genome, are highly enriched in regions with high level of chromatin contact and can be an additional criterion to conduct Hi-C data quality control. Transposable elements have been regarded as noncoding sequences with potential regulatory functions in genomes instead of the orthodoxy “junk DNA” for quite a time. Previous studies of mammalian genomes revealed important functions of TEs, including promoting the evolution of mammalian pregnancy pathway (Lynch et al. 2011; Xie et al. 2010) and tissue-specific transcriptions (Bourque et al. 2008; Faulkner et al. 2009; Lowe et al. 2007; Jin et al. 2012), rewiring the regulatory network in embryo development (Kunarso et al. 2010; Gifford et al. 2013), the microRNA mediated post-transcriptional regulation (Ahn et al. 2013; Berezikov 2011), and the arrangement of CTCF (CCCTC-binding factor) binding sites (Schmidt et al. 2012). The newly studied distal regulatory roles of SINE, the wide-spread retrotransposons in the human genome, are enlightened by chromatin construction capture methods, but only find SINE enriched in regions that separate interaction-rich domains (Lieberman-Aiden et al. 2009; Wang et al. 2011; Eskeland et al. 2010; Kagey et al. 2010). By testing the correlation between different TE types and chromatin interaction levels, we found only SINE but not other TEs enriched in region with high CI scores. Besides, further analysis ruled out non-*Alu* elements in SINE, elucidating that *Alu* element tend to enriched in regions with high CI score for the first time, which suggestion that the *Alu* elements may be significant for distal regulations. High level of chromatin interactions indicates more adjustable transcriptional regulatory events like enhancer-promoter interaction, and has strong nexus with tissue-specific expression (Dixon et al. 2012). There are more than 1 million *Alu* copies in human genome. Besides causing diseases in most time they transpose (Batzer and Deininger 2002; Ule 2013; Winkler et al. 2013; Gallus et al. 2010), the fixed ones often went through selection, and some of them have obtained various important functional roles including inducing enhancers and transcriptional factor binding sites in promoters (Huda et al. 2011; Pastor and Pagani 2011; Antonaki et al. 2011; Cui et al. 2011), deriving alternative spliced exons (Sorek et al. 2002; Shen et al. 2011), and forming alternative promoters, which lead to tissue-specific expression (Faulkner et al. 2009; Lin et al. 2008). Although L1 of LINE, the other active TE in human genome, also can provide source for expression change though mechanisms like generating pseudogenes (Esnault et al. 2000), exonization (Kaer et al. 2011), our results showed that it is *Alu* elements that play the role in distal controlling of genome but not the other TEs, even the other activate ones.

We further suggest *Alu* elements be a new complementary parameter to CI scores on the basis of the significantly positive correlation between the density of *Alu* elements and the score of chromatin interaction (Fig. 2). The Hi-C approach for knowing the genome-wide chromatin contact is undoubtedly remarkable, which makes the search for good ways to eliminate the bias and screen out high-quality pairs important. More than one model was raised to ensure the reliability of Hi-C data, which included procedures like reads quality and length controlling, epigenetic features testing, and controlling GC effects (Yaffe and Tanay 2011; Lu et al. 2013). Considering the significantly high correlation between *Alu* elements and Hi-C data, the *Alu* may also be involved in the Hi-C-normalization models as an important parameter. Association analysis between the amounts of *Alu* and distal regulatory DNA elements like enhancers demonstrated that enhancers are more abundant in regions not only with higher CI scores, but also higher *Alu* coverage (Fig. 4), which indicates that the density of *Alu* can act as an ev*Alu*ator of CI analysis results.

**Figure 5:**
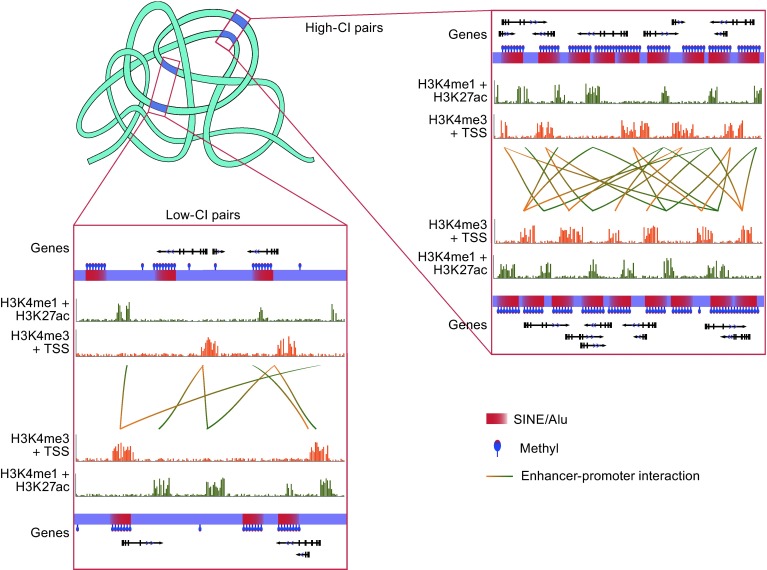
**Different sequence component of regions with high or low CI frequencies**. The right top square represents the high CI region, and the left bottom square represents the low CI region. In each square, the two long purple strips displayed vertically symmetrical demonstrate the two regions interacting with each other. The gradually-changed red squares on the strips show the *Alu* elements with certain direction. The methyl group is represented by a blue ellipse with a red tip on the sequence, which is denser in *Alu* elements than other sequence regions. The active enhancer regions are marked by histone markers H3K4me1 and H3K27ac, and the active TSS are marked by H3K4me3, which are shown by the green and yellow histogram separately. The interaction between enhancer regions and promoters is represented by lines with one end yellow (promoter) and the other arrows

The enhancer sometimes interacts with the promoter distantly and bidirectionally, which is the typical case of distal chromatin interaction that leads to tissue-specific regulation (Banerji et al. 1983; Gillies et al. 1983). Further tests showed that active enhancers regions marked by histone markers H3K4me1 and H3K27ac increased remarkably as the increase of CI frequencies, so as the active TSSs marked by H3K4me3 histone marker (Fig. 4A and 4B). When using the sequence coverage of *Alu* elements as the independent variable, the number of enhancers and promoters also follow the increasing pattern with a correlation *P* value less than 0.05 in both hESC and IMR90 fibroblasts (Fig. 4C and 4D), suggesting that the number of *Alu* elements could act as the one of the measures or filter criteria when estimating genome-wide active enhancer-promoter interaction (Fig. 5). The results above were then confirmed by enhancers of K562 leukemia cells and HeLa cells predicted by ChIP-chip approach and experimental-verified enhancers in a VISTA enhancer browser (Heintzman et al. 2009; Visel et al. 2007). In the investigation, the 40 kb regions with *Alu*-derived enhancers as the center were covered by significantly more *Alu* elements than random sequences, revealing the spatial consistency of *Alu* elements and enhancers. In short, we concluded, for the first time, the indicator potentiality that *Alu* may play in distal chromatin interactions, which is corroborated by the data of two other cell lines from an independent study and a widely-accepted experimental-reviewed database.

### The high GC content in chromatin interaction hotspot is provided by *Alu* elements

*Alu* elements are GC-rich sequences, of which the presence will raise the GC content of certain sequences, provide more CpG sites for methylation, resulting in the remaining of open chromatin status and higher regulatory flexibility. The sequencing step in the Hi-C method for the data we used suffered from the interference of nucleotide composition, also regarded as GC content, which needed to be converted and normalized to limit the effects (Dixon et al. 2012; Yaffe and Tanay 2011). However, although the Hi-C data we investigated have been normalized to rule out GC effect, it still showed a significant correlation with CI scores (Fig. 3). This phenomenon should be caused by functional DNA elements enrichment, which is consistent with other studies. Investigation of the contribution *Alu* elements made to GC content demonstrated that the correlation of *Alu*-provided GC is also positively correlated with CI score, and the pattern is share consistency with the genome background, while the regions not covered by *Alu* elements contribute less GC content to the regions with higher CI scores. The increasing total GC content consisting of increased share of *Alu* regions and decreased share of non-*Alu* regions illustrate clearly that the presence of *Alu* elements raise the GC content in the regions with more chromatin contacts.

The arguments for the intriguing and complicated question why the GC content of *Alu*-surrounded DNA in human genomes is high have lasted for more than a decade. Despite the *Alu* elements making use of the insertion pathway of LINEs, which have a preference for AT-rich integration sites and lead to the accumulation of LINEs in AT-rich region, the human genome project strongly supports that the *Alu* elements tend to be distributed in GC-rich regions (Lander et al. 2001; Jurka 1997). Additionally, further analysis showed that the distribution of “young” *Alu* elements were still in accordance with the pattern of LINE, while the “old” *Alu* elements tended to accumulated in GC-rich regions, indicating the functional role *Alu* may play (Lander et al. 2001). Other studies soon followed up trying to determine the reason why the *Alu* distribution is relative to the GC content (Kunarso et al. 2010; Brookfield 2001), but none of them denied the phenomenon that most *Alu* elements, especially the old ones, have a surrounded area with high GC content. As expected, our results also proved the old finding that there are more *Alu* elements displayed in regions with higher GC content. However, it seemed that it is the GC-rich *Alu* elements themselves raise the GC content of the whole region, but not the sequences not belonging to *Alu*s. That is to say, the regions with high CI frequencies own more *Alu*s that have more guanine and cytosine, and the whole GC content of these regions are higher than the regions with less chromatin contact as a result (Fig. 5). To summarise, the finding that the *Alu* contributes more GCs in regions with higher CI scores combined and answered the two long controversial questions together, pointed out that the *Alu* elements are the reason why active interacted region have a higher GC content, and it is the *Alu* elements gathered together that raise the GC content of the region, not only the surrounding DNA but also itself.

The amount of GC can affect the chromatin structure directly, while a large part that forms its biological function is that DNA can be methylated at CpG sites, which will accumulate when there is more GC as our results show (Fig. S5). By comparing the genome-wide CpG density in different part of the human genome, we found that the although the CpG density of each region showed positive correlation with CI score regardless of whether the sequences are covered by *Alu* elements, the higher the CI score is, the more CpG comes from *Alu* elements than non-*Alu* regions (Fig. 3B), which indicates that *Alu* elements not merely generate GC content rise in regions with high CI score, they also provide more CpG sites that can be methylated. When we looked into the proportion of how much the GC content becomes CpG sites, the results showed that the increasing GC content and CpG sites are connected with the growth in *Alu* number, but not the changing of the *Alu* sequences (Fig. 3C). The results shown above highlight again that the more *Alu* elements in regions with more chromatin contact can provide additional GC content as well as CpG sites.

The CpG sites in vertebrates are known for typical DNA methylation. The methylation process at CpG sites is by adding a methyl group to cytosine and turning it to 5-methylcytosine, which may change the structure of chromatin and result in various biological consequences. For example, the methylation surrounding TSS will block the initiation of transcription, while the methylation in the gene body will not block the transcription but even sometimes stimulate it (Suzuki and Bird 2008). The TEs are often highly methylated since most transposable events are deleterious, and the host genome must try to suppress the activation of TEs (Yoder et al. 1997; Bestor 1998). In our results, the *Alu*s showed a very high level of methylation in the whole genome with more than 90% of the CpG sites in *Alu* regions methylated regardless the cell types (Fig. S5C and S5D). This is consistent with the previous studies, and elucidated clearly that the transposition of *Alu* is not welcomed by genome and should be under control. The methylation rate showed a slightly negative correlation in hESC cell line, which maybe result from the several abnormal values at the high-CI regions, and it is not correlated with CI correlation in IMR90 fibroblasts. The high methylation in *Alu* elements also raises the methylation level of promoters and gene bodies when *Alu* is inserted into genes (Fig. S7C–F), which is quite reasonable because the transposition of *Alu* elements in actively transcribing genes should be strictly restricted. Nevertheless, the promoter region without *Alu* elements possessed more percentage of methylated CpG sites in higher-CI regions. Obviously, the promoters in regions with more chromatin contacts are actively regulated by DNA elements, so they have loose chromatin structures with less methylation. But the significant decreasing pattern of methylation did not happen in the gene body, which only had a weak decreasing trend as the CI score increased. As mentioned above, the methylation in CpG sites will not affect the transcription in the gene body, sometimes even promote it, which make our results rational no matter where the gene is actively regulated of trancribing, the methylation density in the gene body will not change much. Moreover, the methylation density in the gene body is always higher than the promoter as our results demonstrate (Fig. S7A and S7B), which confirmed again that the CpG islands are barely methylated when located around transcription start sites (TSSs), but will be methylated when in gene bodies in a tissue-specific manner.

### Different *Alu* subfamilies follow the chronological methylation

*Alu* elements were noticed to have the GC-rich region preference after the human genome was sequenced, with the “old” ones tending to accumulate in GC-rich regions and “young” ones AT-rich regions, (Lander et al. 2001). Here we tested the methylation rate of the CpG sites in *Alu* elements with different ages, and found that besides the difference in preference for GC content, the “old” *Alu* elements were more highly methylated than the “young” ones (Fig. 3D). Those young *Alu* elements are generally more transcriptionally active than old ones results from various reasons. One of the reasons is the accumulation of mutations in *Alu* elements disables the recognition of transposase; others include methylation and RNA interferance by the host genome, which are more important. The mutations will accumulate as time passes, so the older ones must contain more mutated sites; however the methylation status will not always become increasingly intensive. The host genome cannot recognize new *Alu* repeats immediately after insertion, and forbid its future transposition with methylation, so the young *Alu*Y may have less methylated CpG sites and have less mutations in key regions related to transposition as our results indicate. As the *Alu* elements stay in the genome for more time, the host genome will discover this sequence and methylate it more often, and the sequence will change at a certain rate itself. Under the pressure of evolution, the methylation of CpG will eventually reach saturation or just enough to suppress the move and remain stable, and the mutation will not stop. When the sequence is converted so much that it could not be identified by transposase and will be silent forever, the deleterious nature of the *Alu* is fully removed, and the host genome may release it from its high methylation status as time goes by. According to our investigation of old *Alu*J, median *Alu*S and young *Alu*Y subfamilies, the *Alu*J have a slightly higher methylation density than *Alu*S, and both of them are almost fully methylated with more than 90% CpG sites methylated, while only less than 80% CpG sites in *Alu*Y were methylated. The average age of *Alu*J and *Alu*S is 81 million years and 36 million years (Kapitonov and Jurka 1996), while *Alu*Y sequences are very young and still fully active in human genome. If the methylation rate of the host genome is stable, we can estimate that the methylation rate of CpG sites in *Alu* elements is about 0.004% per million-year using the methylation density in our result, which means in another 10 million years, all the *Alu*Y will be fully methylated and silent as the presents *Alu*J. However, the new insertions will still be active and may form another subfamily that can still affect the host genome in various ways.

## CONCLUSIONS

The *Alu* elements are highly enriched in the genome region with high levels of chromatin interaction. Correlation analysis reveals that the sequence character of *Alu* elements and its enrichment have endowed the actively regulating regions with a higher GC content, together with more CpG sites, and also a higher methylation density. The increase in methylation potentiality will provide flexibility to the sequences and transcriptional regulation as shown in Fig. 5. That is why the high CI frequencies always relate to tissue-specific expression. In contrast, other TEs including the active L1 in human genome are not enriched in high-CI regions and show a random distribution when evaluated by CI frequency.

## MATERIALS AND METHODS

### Processing chromatin interaction data

The recently released genome-wide chromatin interaction profiling of two cell lines in human, includes human embryonic stem (ES) cells and human IMR90 fibroblasts corresponding to pluripotent and differentiated cells. In these two cell lines, the datasets with the bin size of 40 kilobases (kb) were adopted, since chromatin interaction (CI) frequencies were normalized for biases in the data. The coordinates of domain-boundary annotations were converted from Hg18 to Hg19 using the UCSC Genome Browser liftover utility (http://genome.ucsc.edu/cgi-bin/hgLiftOver). We discarded the domains and bins, of which coordinates failed to remap, which is about 6.68% of the bins of Hg18. We then calculated the mean inter-domain CI frequencies in each cell line and used these values as the lower threshold for each cell line (3.34 for hESC and 3.06 for IMR90) and discarded all the bin-pairs whose CI frequencies are lower than the threshold in the corresponding cell lines (assuming they are non-informative or background noises in hESC and IMR90 cell lines). We then calculated the number of bin-pairs to be filtered out by setting upstream thresholds increased from the downstream threshold by 1 as the step each time, and finally removed the bin-pairs whose CI frequencies are larger than 153 in hESC and 133 in IMR90, respectively (assuming they are outlier in each cell line). Finally, we were able to retain 897,867 bin-pairs for hESC and 850,305 bin-pairs for IMR90.

### Compiling TEs

The coordinates of TEs in the human genome were downloaded from RepeatMasker (http://www.repeatmasker.org, release version 3.3.0, Repbase library version 20110920) (Hubley and Green 1996). Based on the classification of RepeatMasker program, we divided all the TEs to four major types (LINE, SINE, LTR, and DNA) and discarded the TEs with uncertain categories. The coverage of certain TE type or TE family was defined as the number of nucleotide in the chromatin interaction bin-pairs by using the length and annotation of TEs. For the *Alu* subfamilies, *Alu*J, *Alu*S, and *Alu*Y, we counted the number of the TE copies of certain *Alu* subfamilies in chromatin interaction bin-pairs regarded as the enrichment score. The reason why we used coverage for four TE types, but used enrichment score for *Alu* subfamilies is that the average lengths of the four TE types are different, while those of the *Alu* subfamilies are nearly the same. The coverage of TE copies and the enrichment score of *Alu* subfamilies in bin-pairs with different CI frequencies were then used in the Pearson correlation coefficient analysis with other parameters.

### Determination of the methylation density

The coordinates of methylated CpG sites of both hESC cell line and IMR90 fibroblasts were based on the results of recent work (http://neomorph.salk.edu/human_methylome) (Lister et al. 2009) and were converted from Hg18 to Hg19 by using UCSC Genome Browser liftover utility (http://genome.ucsc.edu/cgi-bin/hgLiftOver). To evaluate the methylation level, we calculated the percentage of methylated sites of all the CpG sites in each bin-pair, termed methylation density. Similarly, the CpG density was defined as the percentage of CpG sites in all the C/G nucleotide in each bin-pair, while the GC content is the percentage of C/G nucleotide in all the nucleotide in each bin-pair.

### Identification of active genes and cis-elements

The active genes were identified in both hESC cell lines and IMR90 fibroblasts by combining the gene annotation and histone markers. We used annotated mRNAs in RefSeq genes and UCSC genes, and de-redundant by keeping the longest gene loci in gene loci with the same gene symbols. The ChIP-Seq data of H3K4me1, H3K27ac, and H3K4me3 histone markers were downloaded from GEO database (GSE16256), and processed by Model-based Analysis for ChIP-Seq (MACS) to rule out the false positive peaks with the false discovery rate (FDR) value over 5% (Heintzman et al. 2009; Zhang et al. 2008; Hawkins et al. 2010). We then combined the processed transcription start sites (TSS) annotation and H3K4me3 peaks to obtain the active markers. The active enhancer regions were defined by the overlapping regions of H3K4me1 and H3K27ac markers. Active TSSs were defined by H3K4me3 peaks on TSS. If a gene locus has multiple TSS, we choose the TSS with most H3K4me3 peak as the promoter of the whole gene locus. To evaluate the difference between enhancer regions and TSS regions in two different cell lines, we regarded the enhancers or promoters from two datasets a same one if the distance between two peaks is less than 250 bp. The hESC and IMR90 groups share 2126 enhancers, which are 8.2% in hESC and 7.2% in IMR90. The hESC and IMR90 groups share 5292 TSSs, which are 80.7% in hESC and 83.8% in IMR90 (Fig. S9A).

### Correlation analysis between TEs and other chromatin characters

The PCC between the coverage of different TE families and the CI frequencies in each bin-pairs were calculated. The number of TE copies of *Alu* subfamilies and the CI frequencies were also evaluated by PCC. The number of GC content, CpG content and methylation density were investigated by PCC with CI frequencies in different regions in each bin-pairs including regions covered by *Alu* elements, regions covered by SINE elements but not *Alu*, and regions not covered by SINE. The PCC between the number of promoters and enhancers annotation by histone markers and the CI frequencies were calculated.

### Processing enhancer data of K562 and HeLa cell lines, and VISTA database

The predicted enhancers of K562 and HeLa cell lines were adopted from Bing Ren’s study (Heintzman et al. 2009). We also downloaded the reviewed data in the VISTA enhancer browser (http://enhancer.lbl.gov/frnt_page_n.shtml). We then grouped all the enhancers to *Alu*-derived class and non-*Alu*-derived class depending on whether their peaks are in *Alu*s, and then calculated the mean *Alu* coverage in 20 kb upstream to 20 kb downstream in the two classes. We evaluated the enhancers in three datasets by comparing the distance between their peaks. If the distance between two peaks is less than 250 bp, we regarded them as a same enhancer. The three groups had very different patterns that K562 and HeLa groups share 1200 enhancers, which are 3.3% in HeLa and 4.9% in K562. The K562 and Vista groups share 22 enhancers, which are 0.09% in K562 and 2.7% in Vista data. The HeLa and Vista groups share 28 enhancers, which are 0.08% in HeLa and 3.5% in Vista data. And only 4 enhancers are shared by all three groups, which are 0.01% in HeLa, 0.02% in K562, and 0.5% in Vista data (Fig. S9B).

## Electronic supplementary material

Below is the link to the electronic supplementary material.

Supplementary material 1 (XLSX 60 kb)

Supplementary material 2 (PDF 1513 kb)
