# Supplementary material for: Enrichment analysis of Alu elements with different spatial chromatin proximity in the human genome
Source: Protein Cell. 2016 Feb 10;7(4):250–66. doi: 10.1007/s13238-015-0240-7 (PMC4818845; doi:10.1007/s13238-015-0240-7)
Supplement: Supplementary file 2 — Supplementary material 2 (PDF 1513 kb) [file 13238_2015_240_MOESM2_ESM.pdf]

Supplementary Figure legend

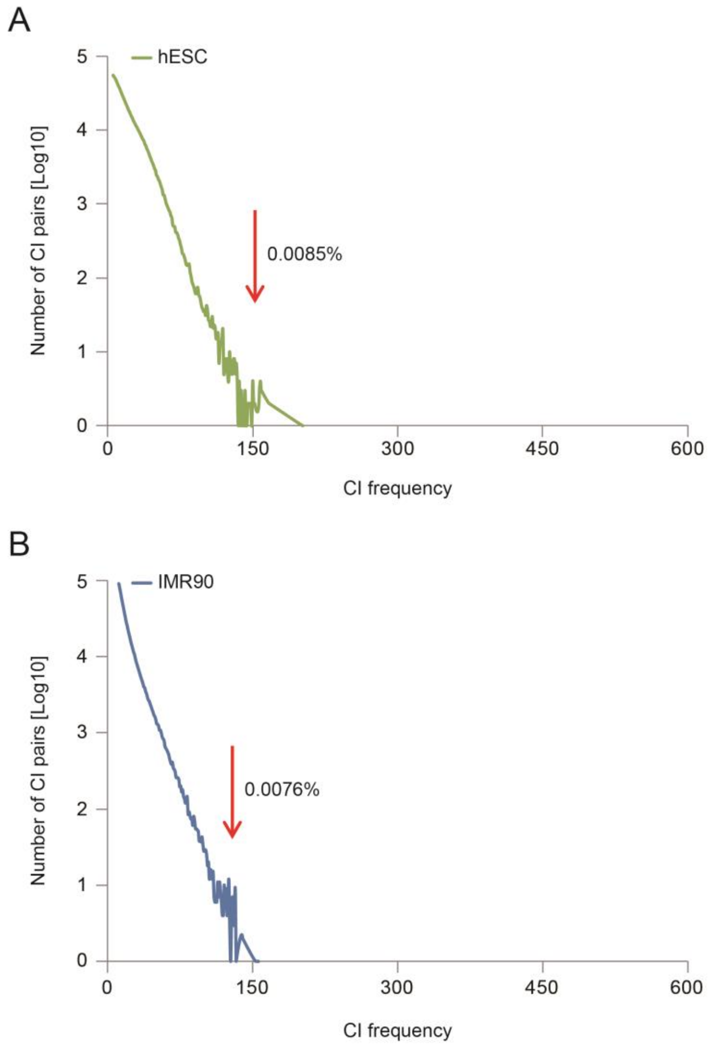

Figure S1: Distribution of CI pairs at each CI frequency (bin size = 1)

Figure S1

Figure S1

Distribution of CI pairs at each CI frequency (bin size = 1)

A. The number of bin-pairs decreased sharply with the increasing of CI frequencies and was discrete at 153 in hESC.

B. The CI frequency when bin-pair became to discrete is 133 in IMR90.

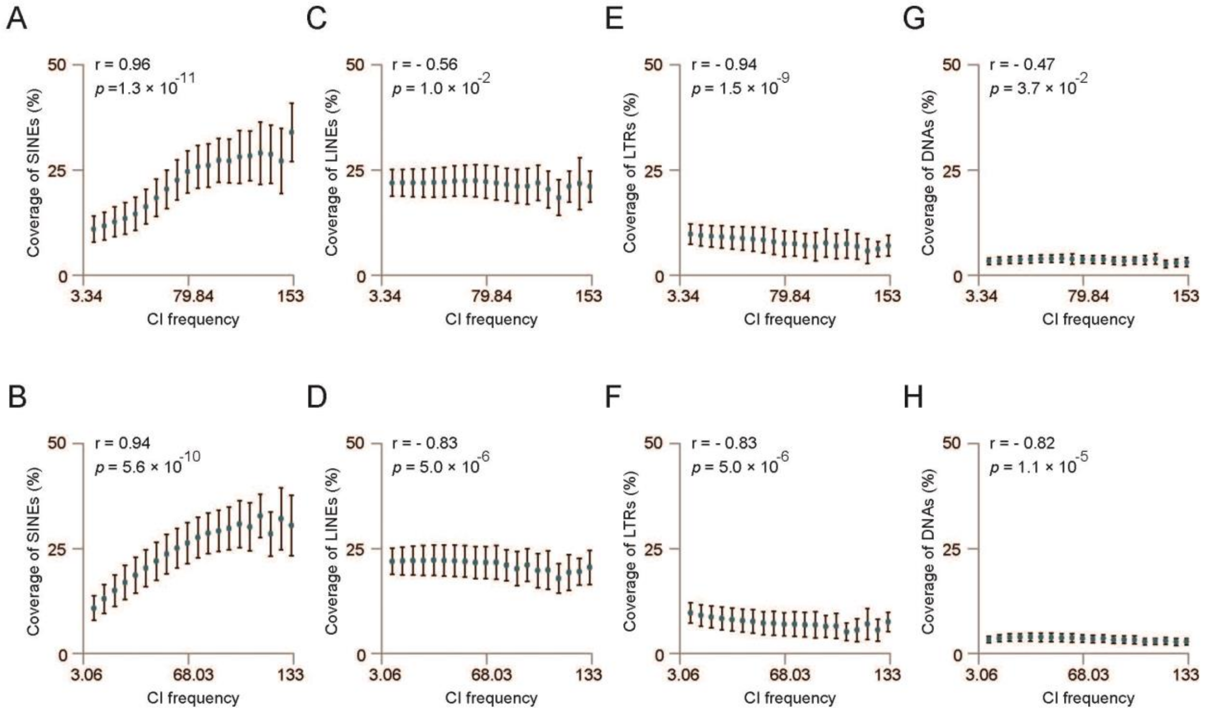

Figure S2

Figure S2

Mean coverage (MC) of four TE family types in each 20 frequency bin. In both cell lines, the SINE is the only TE type of which the coverages in bin-pairs were positively correlated with CI frequencies, while the other three negatively correlated.

A: the MC of SINE in 20 frequency bins in hESC. B: the MC of SINE in 20 frequency bins in IMR90.

C: the MC of LINE in 20 frequency bins in hESC. D: the MC of SINE in 20 frequency bins in IMR90.

E: the MC of LTR in 20 frequency bins in hESC. F: the MC of SINE in 20 frequency bins in IMR90.

G: the MC of DNA in 20 frequency bins in hESC. H: the MC of SINE in 20 frequency bins in IMR90.

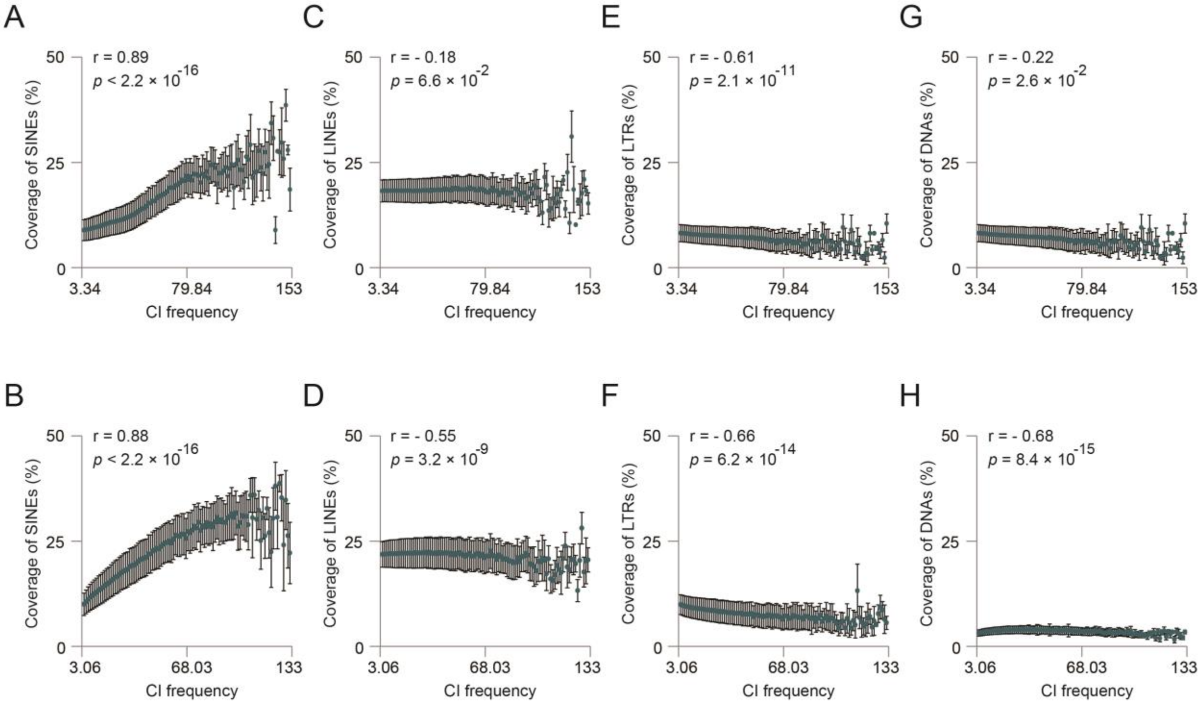

Figure S3

Figure S3

Mean coverage (MC) of four TE family types in each 100 frequency bin

In both cell lines, the SINE is the only TE type of which the coverages in bin-pairs were positively correlated with CI frequencies, while the other three negatively correlated.

A: the MC of SINE in 100 frequency bins in hESC. B: the MC of SINE in 100 frequency bins in IMR90.

C: the MC of LINE in 100 frequency bins in hESC. D: the MC of SINE in 100 frequency bins in IMR90.

E: the MC of LTR in 100 frequency bins in hESC. F: the MC of SINE in 100 frequency bins in IMR90.

G: the MC of DNA in 100 frequency bins in hESC. H: the MC of SINE in 100 frequency bins in IMR90.

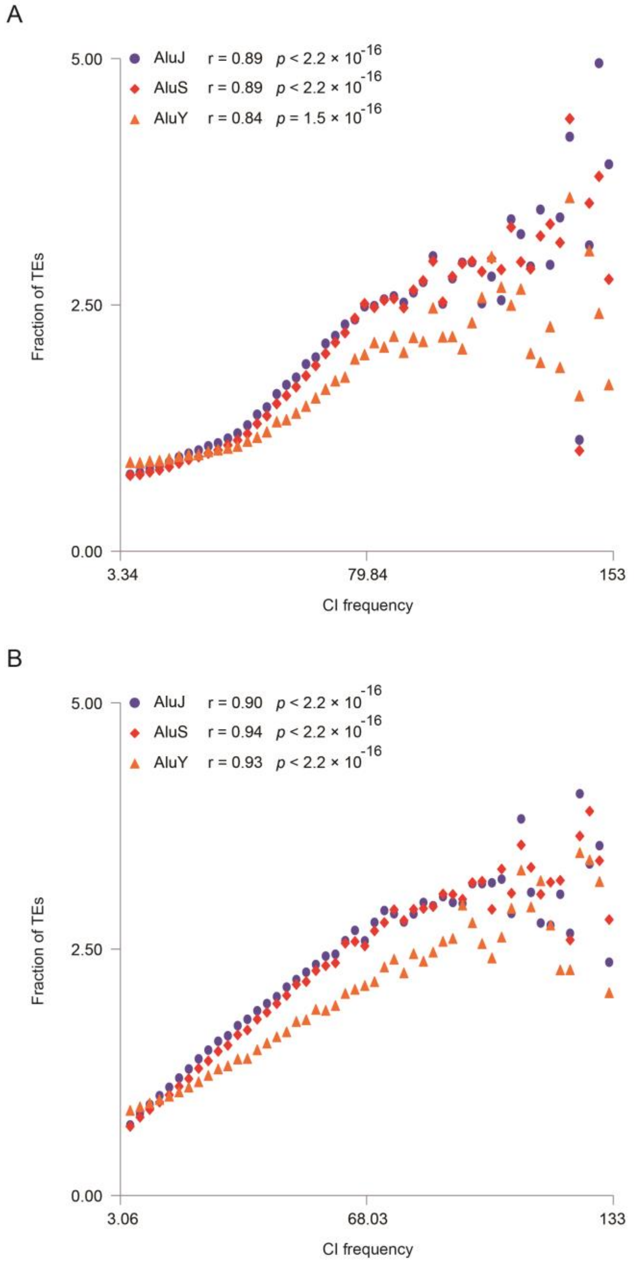

Figure S4

Figure S4

Mean coverage (MC) of three *Alu* subfamilies in each 50 frequency bin (A. hESC; B. IMR90 fibroblasts). All the three subfamilies showed positive correlation with CI frequencies.

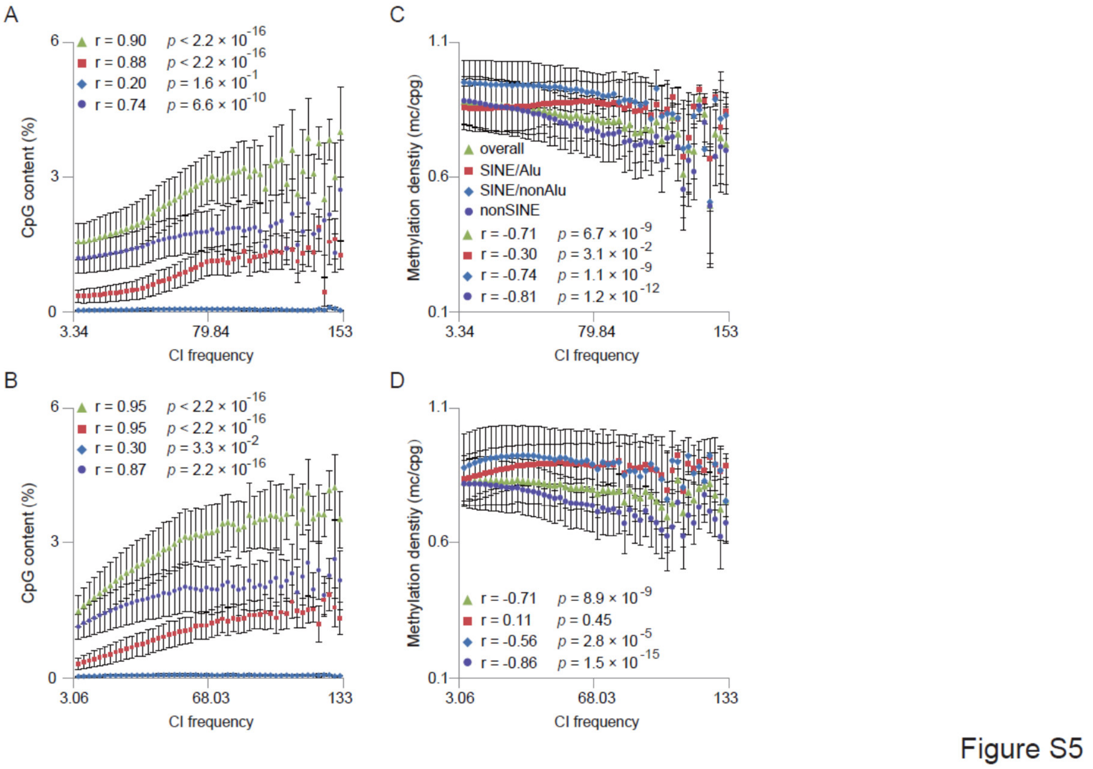

Figure S5

The methylation density of bin-pairs.

A-B: the methylation of genome background, *Alu* elements regions, SINE/non-*Alu* regions (other transposable elements in SINE type) and non-SINE regions (regions that don't covered by SINEs) in each frequency bin (A:hESC; B: IMR90 fibroblasts)

C-D: the methylation rate of CpG sites of the methylation of genome background, *Alu* elements regions, SINE/non-*Alu* regions and non-SINE regions in each frequency bin.

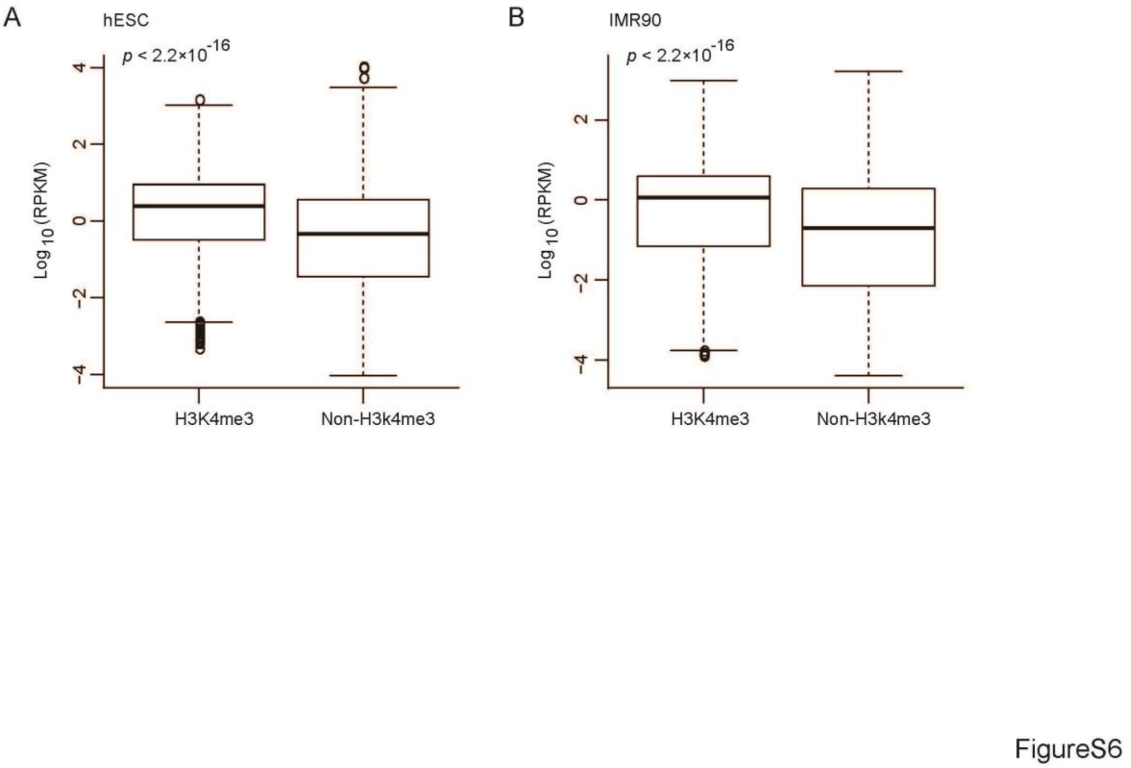

Figure S6

The expression level of genes with TSS marked/not marked by H3K4me3.

A: The boxplot showed the distribution of RPKM of genes with TSS. The p-value of T-test showed the difference between the expression levels of genes with TSS marked/not marked by H3K4me3 in hESC.

B: The difference between the expression levels of genes with TSS marked/not marked by H3K4me3 in IMR90.

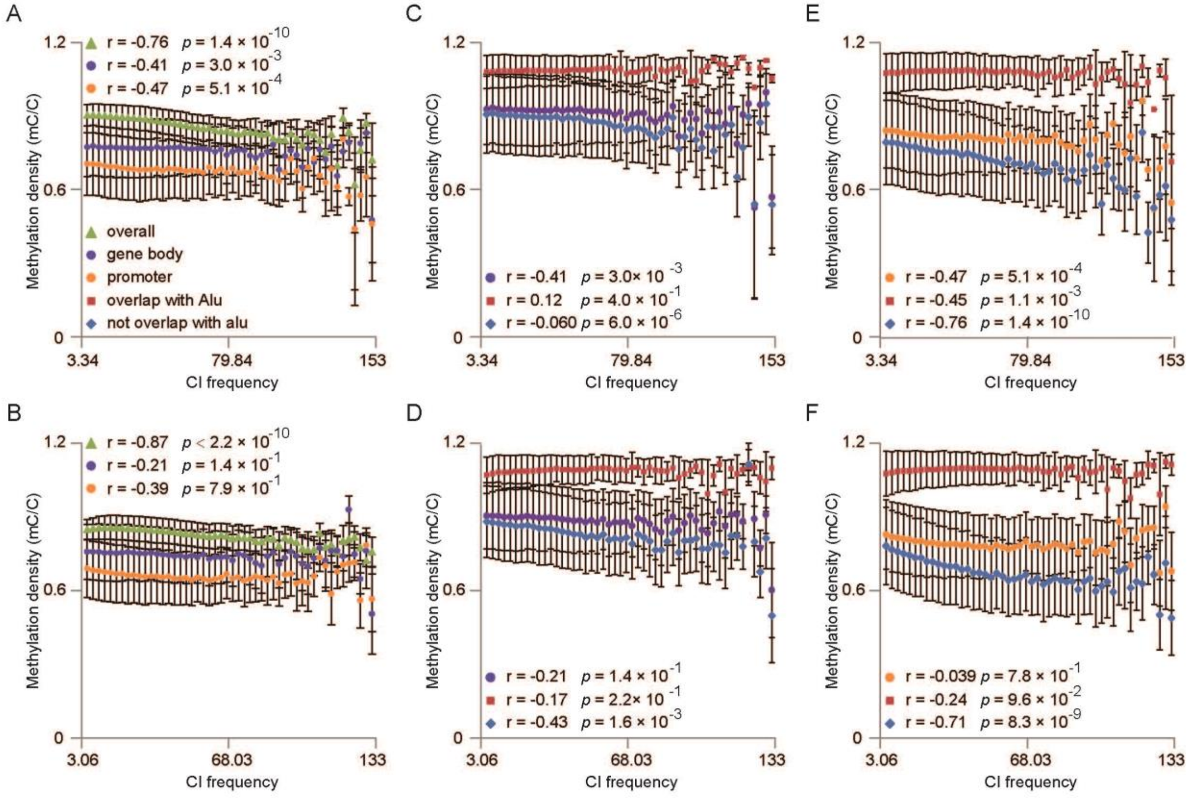

Figure S7

Figure S7

The methylation densities of gene bodies and promoters with different CI frequencies in both hESC cell lines and IMR90 fibroblasts.

A-B: The methylation density of genome background was higher than that of gene bodies, followed by promoter regions (A. hESC; B. IMR90 fibroblasts).

C-D: When divided by *Alu*-covered regions and non-*Alu*-covered regions, the *Alu*-covered gene body had a high methylation density and the non-*Alu*-covered gene body had a comparatively low methylation density (C. hESC; D. IMR90 fibroblasts).

E-F: When divided by *Alu*-covered regions and non-*Alu*-covered regions, the *Alu*-covered promoter regions had a high methylation density and the non-*Alu*-covered promoter regions had a comparatively low methylation density (E. hESC; F. IMR90 fibroblasts).

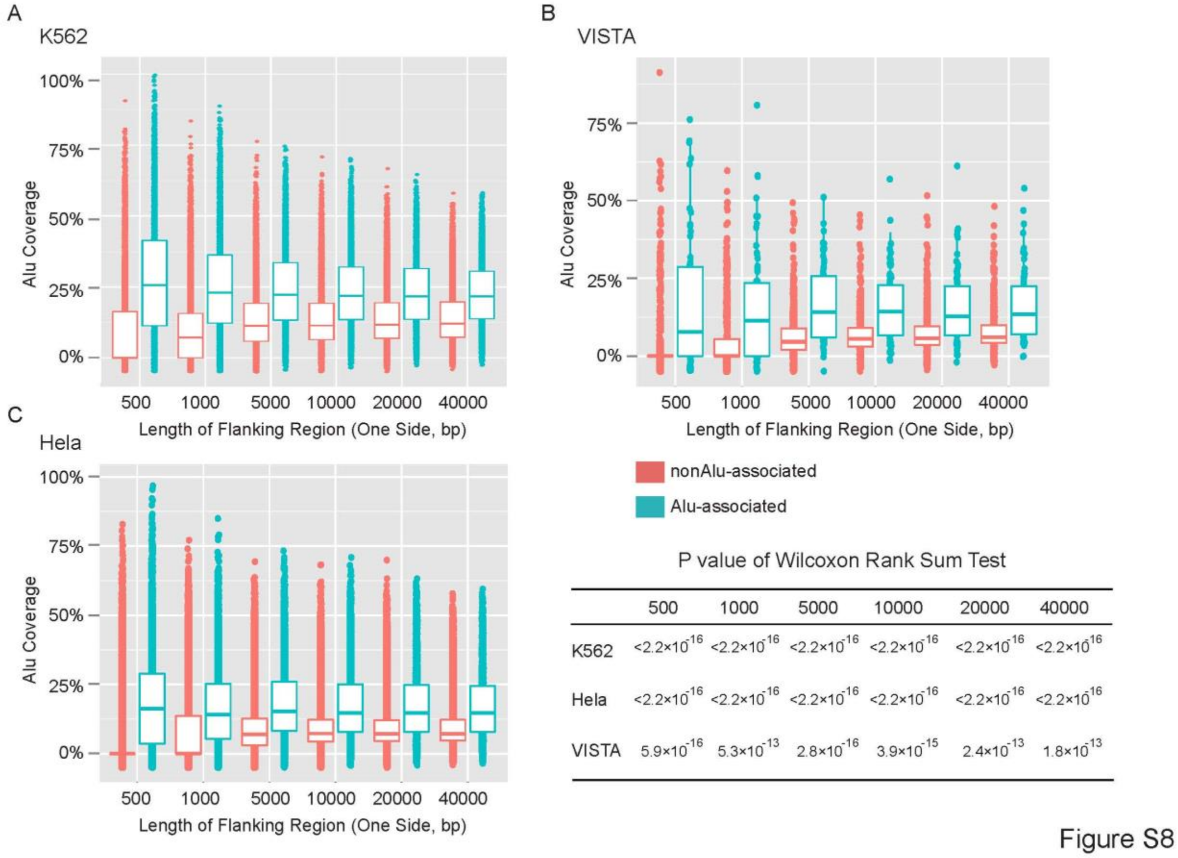

P value of Wilcoxon Rank Sum Test

	500	1000	5000	10000	20000	40000
K562	$<2.2\times10^{-16}$	$<2.2\times10^{-16}$	$<2.2\times10^{-16}$	$<2.2\times10^{-16}$	$<2.2\times10^{-16}$	$<2.2\times10^{-16}$
Hela	$<2.2\times10^{-16}$	$<2.2\times10^{-16}$	$<2.2\times10^{-16}$	$<2.2\times10^{-16}$	$<2.2\times10^{-16}$	$<2.2\times10^{-16}$
VISTA	5.9×10^{-16}	5.3×10^{-13}	2.8×10^{-16}	3.9×10^{-15}	2.4×10^{-13}	1.8×10^{-13}

Figure S8

The Alu coverage of flanking regions (one side: 500bp, 1kb, 5kb, 10kb, 20kb, 40kb) of Alu associated/nonAlu-associated enhancers.

A: Enhancers predicted by Histone marks in K562 cell line.

B: Enhancers reviewed by experimental verification in VISTA enhancer browser.

C: Enhancers predicted by Histone marks in Hela cell line.

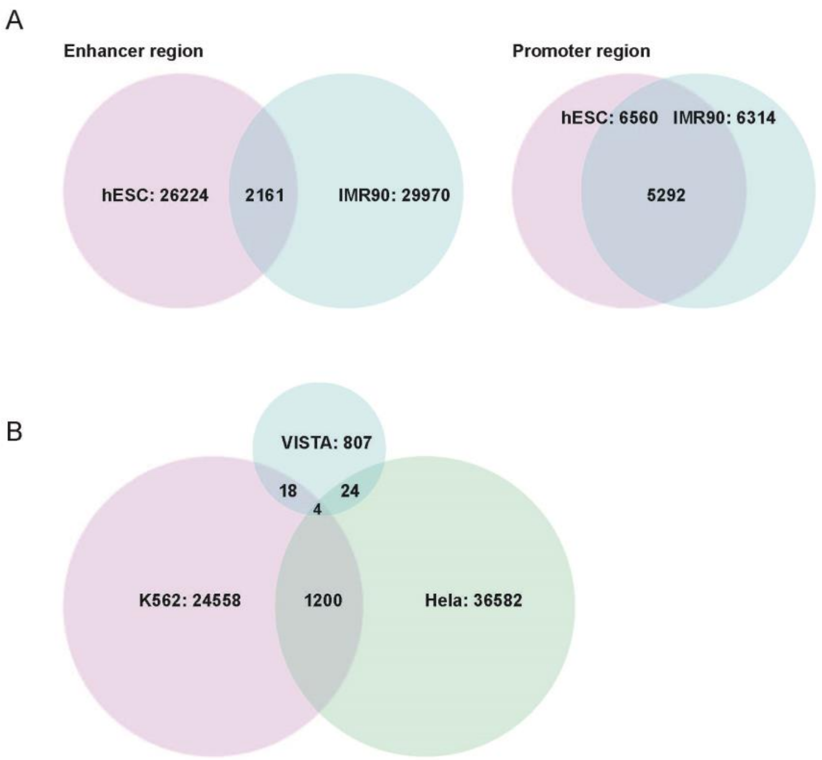

Figure S9

Figure S9

The similarity of enhancer datasets and TSS datasets.

A: The similarity of predicted enhancers and TSS in hESC and IMR90 fibroblast.

B: The similarity of predicted enhancers in k562, hela and experimental reviewed enhancers from VISTA enhancer browser.
